# Curcumin and (−)- Epigallocatechin-3-Gallate Protect Murine MIN6 Pancreatic Beta-Cells against Iron Toxicity and Erastin-Induced Ferroptosis

**DOI:** 10.3390/ph12010026

**Published:** 2019-02-06

**Authors:** Tugba Kose, Mayra Vera-Aviles, Paul A. Sharp, Gladys O. Latunde-Dada

**Affiliations:** Department of Nutritional Sciences, School of Life Course Sciences, King’s College London. Franklin-Wilkins-Building, 150 Stamford Street, London SE1 9NH, UK; tugba.kose@kcl.ac.uk (T.K.); mayra.vera_aviles@kcl.ac.uk (M.V.-A.); paul.a.sharp@kcl.ac.uk (P.A.S.)

**Keywords:** curcumin, (-)-epigallocatechin-gallate, iron, ferroptosis

## Abstract

Ferroptosis is a form of programmed cell death that is characterized by lipid peroxidation and is inducible by iron and the accumulation of reactive oxygen species (ROS). It is triggered by erastin but inhibited by antioxidants such as α-tocopherol, β-carotene, polyphenols, and iron chelators such as deferoxamine (DFO), nitrilotriacetic acid (NTA), and ethylenediaminetetraacetic acid (EDTA). This study investigated the protective effects of two polyphenols, curcumin and (−)- epigallocatechin-3-gallate (EGCG), against iron loading and erastin-mediated ferroptosis in MIN6 cells. Cells were treated with polyphenols before exposure to iron-induced oxidative stress comprising of 20 μmol/L of 8-hydroxyquinoline (8HQ) and 50 μmol/L of ferric ammonium citrate, (FAC) (8HQ+FAC) or Fenton reaction substrate (FS) (30 μmol/L of FeSO_4_ and 0.5 of mmol/L H_2_O_2_) and 20 μmol/L erastin. Cell viability was determined by 3-(4,5-dimethyl-2-thiazolyl)-2,5-diphenyltetrazolium bromide (MTT) assay, iron levels were measured by inductively-coupled plasma mass spectrometry (ICP-MS), glutathione and lipid peroxidation were assayed with commercially-available kits. Curcumin and EGCG both significantly protected pancreatic cells against iron-induced oxidative damage. Moreover, both compounds also protected against erastin-induced ferroptosis in pancreatic cells. The polyphenols enhanced cell viability in erastin-treated MIN6 cells in a dose- and time-dependent manner. Furthermore, MIN6 cells exposed to erastin alone showed elevated levels of iron, glutathione (GSH) depletion, glutathione peroxidase 4 (GPX4) degradation and lipid peroxidation (*p* < 0.05) compared to cells that were protected by pre-treatment with curcumin or EGCG. Taken together, the data identify curcumin and EGCG as novel ferroptosis inhibitors, which might exert their protective effects by acting as iron chelators and preventing GSH depletion, GPX4 inactivation, and lipid peroxidation in MIN6 cells. The implications of the findings on the effects of iron overload and ferroptosis represent a potential therapeutic strategy against iron-related diseases.

## 1. Introduction

Iron is a vital trace metal in physiological processes such as oxygen transport, respiration, energy generation and DNA synthesis, and is a structural component of numerous enzymes and proteins in the body. The ability of iron to perform these functions relies on its existence in variable and interconvertible oxidation states. Iron can exchange single electrons with metabolites to generate reactive oxygen species (ROS) that are capable of causing DNA damage, protein denaturation, and lipid peroxidation [1]. Together, these events have recently been defined as a form of programmed cell death called ferroptosis [2]. Excessive ROS production is neutralized by a repertoire of defence antioxidants enzymes such as superoxide dismutase, catalase, and glutathione peroxidases (GPXs) [3]. Dysregulation of iron homeostasis can lead to iron-overload disorders such as hemochromatosis and thalassemia, which are characterized by elevated levels of ROS. It is noteworthy that there is a high prevalence of type 2 diabetes mellitus (T2D) amongst patients [4], and epidemiological studies have reported significant associations between excess iron stores and T2D [5,6]. Toxicity of iron in pancreatic β-cells, possibly because of elevated ROS, results in decreased insulin synthesis and secretion [6]. Iron overload-mediated oxidative stress causes impaired insulin signalling and inhibition of ATP production due to mitochondrial dysfunction [6,7]. Furthermore, reduced antioxidant levels in conjunction with increased free radical production have been reported to cause pancreatic β-cell damage [8,9].

Although synthetic iron chelators such as deferoxamine (DFO), nitrilotriacetic acid (NTA) and ethylenediaminetetraacetic acid (EDTA) are used in treating secondary iron overload disorders, in particular natural plant products or bioactive compounds of plant origin such as polyphenols are emerging as potent and efficacious alternatives [10,11]. Moreover, in addition to their iron chelating properties, some polyphenols also possess antioxidant potential [12]. Polyphenols such as curcumin and (−)- epigallocatechin-3-gallate (EGCG) have been shown to form complexes readily with different metal ions [12,13].

Ferroptosis emerged recently as a phenomenon that encapsulates the interplay of iron accumulation, excessive ROS levels and lipid peroxidation, and culminates in cell death that is associated with degenerative disorders [14]. In light of this, the current study investigated the protective functions of polyphenols against iron toxicity and erastin-induced ferroptosis in murine MIN6 cells. In essence, the investigation was designed to demonstrate the inhibition of erastin-induced ferroptosis, iron accumulation, and lipid peroxidation by curcumin and EGCG inclusion in treated MIN6 pancreatic cells.

## 2. Results

### 2.1. The Protective Effects of Polyphenols on Iron-Induced Oxidative Stress

**Figure 1:**
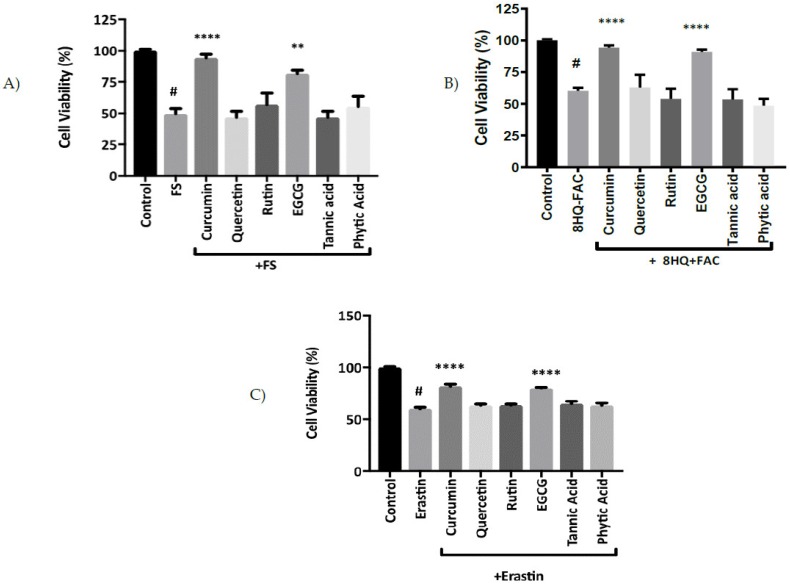
Protective effects of different polyphenols against iron-mediated toxicity and ferroptosis. MIN6 cells were treated with 20 μM curcumin, quercetin, rutin, EGCG, tannic acid or phytic acid for 24 h and supplemented with (**A**) Fenton Substrates (FS), (**B**) 8HQ+FAC for 2 h and (**C**) MIN6 cells were treated overnight with 20 μM erastin, in the absence or presence of quercetin, rutin, curcumin, tannic acid, phytic acid and EGCG. The percentage of cell viability is relative to control cell samples. Curcumin and EGCG inhibited erastin-induced cell death in a dose-dependent manner. Curcumin and EGCG had a protective effect against ferroptosis at 20 μM in MIN6 for 24 h, with a valuable statistical difference between erastin and cell treated with erastin + 20 μM curcumin or EGCG. All the values are expressed with the mean ± SEM, *n =* 8. ^#^*p* < 0.05 control vs. treatment groups, **p < 0.01 and ****p < 0.0001 compared with FS and 8HQ+FAC group only. (**C**) ^#^*p* < 0.05 control vs. treatment groups, ****p < 0.0001 vs. erastin only. One-way ANOVA, Tukey post-hoc test.

Initial experiments investigated the sensitivity of MIN6 pancreatic cells to iron-induced stress and the protective effects of polyphenolic compounds. In cells pre-incubated with six different polyphenols before exposure to FS and 8HQ+FAC for 2 h, only curcumin and EGCG showed significant protection against iron-induced cell damage (Figure 1A,B).

### 2.2. Protective Function of Curcumin and EGCG against Ferroptosis

Having identified curcumin and EGCG as the most potent polyphenols for conferring protection against iron-induced stress, we next investigated the potential of these compounds to act as inhibitors of ferroptosis. Subsequently curcumin and EGCG were compared with quercetin, rutin, tannic acid and phytic acid as inhibitors of erastin-induced ferroptosis in MIN6 cells. Of the six compounds that were tested, curcumin and EGCG exhibited the best protection against erastin-induced cell death in MIN6 cells (Figure 1C).

### 2.3. Dose-Response Effects of Curcumin and EGCG against Erastin-Induced Ferroptosis 

**Figure 2:**
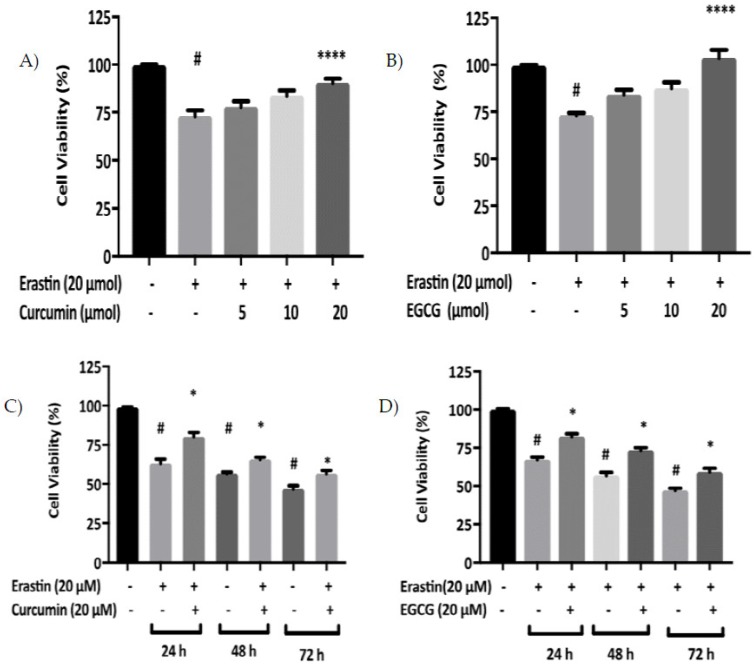
Anti-ferroptosis activity of curcumin and EGCG in MIN6 cells. Cells were treated overnight with 20 μM erastin in the absence or presence of curcumin or EGCG. The percentage of cell viability is relative to control cell samples. Curcumin and EGCG inhibited erastin-induced cell death in a dose-dependent manner. Curcumin (**A**) and EGCG (**B**) had a protective effect against ferroptosis at 20 μM in MIN6 for 24 h, with valuable statistical difference between erastin and cell treated with erastin + 20 μM curcumin or EGCG. Curcumin (**C**) and EGCG (**D**) inhibited erastin-induced cell death in a time-dependent manner in MIN6 with valuable statistical difference between erastin and cell treated with erastin + 20 μM curcumin or EGCG. All the values are expressed with the mean ± SEM, *n =* 8, ^#^*p* < 0.05 control vs. treatment groups, *p < 0.05 and ****p < 0.0001 vs. erastin only. One-way ANOVA, Tukey post-hoc test.

Cell viability was evaluated in cells exposed to a range of EGCG and curcumin concentrations in the presence of erastin for 24 h. Curcumin caused a decrease in cell mortality in a dose-dependent manner, over a concentration range of 5–20 μM in MIN6 cells (Figure 2A). Likewise, EGCG also inhibited erastin-induced cell death in a dose-dependent manner in MIN6 cells (Figure 2B). 

### 2.4. Time Course Effects of Curcumin and EGCG against Erastin-Induced Ferroptosis

Next, we determined the temporal range of protection conferred by curcumin or EGCG against erastin-induced ferroptosis. Protection was evident with both curcumin and EGCG in MIN6 cells after 24 h. Over these specific time points, both polyphenols provided a similar pattern of protection against erastin-induced cell death in MIN6 cells (Figure 2C,D). 

### 2.5. Curcumin and EGCG Limit Iron Accumulation and Lipid Peroxidation in Ferroptosis

**Figure 3:**
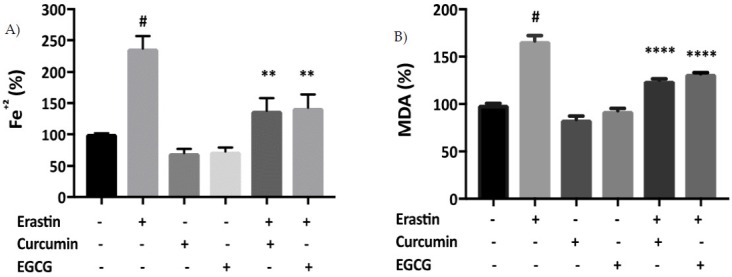
Curcumin and EGCG suppress iron and lipid accumulation in pancreatic cells. (**A**) Cells were treated overnight with 20 μM erastin in the absence or presence of curcumin or EGCG. Percentage of Fe^2+^ is relative to control cell samples. Curcumin and EGCG decreased erastin-induced iron accumulation at 20 μM in MIN6 for 24 h, with significant statistical difference between erastin and cell treated with erastin + 20 μM curcumin or EGCG. (**B**) Percentage of MDA is relative to control cell samples. Curcumin and EGCG decreased erastin-induced lipid peroxidation at 20 μM in MIN6 for 24 h, with valuable statistical difference between erastin and cell treated with erastin + 20 μM curcumin or EGCG. All the values are expressed with the mean ± SEM, *n =* 3, *^#^p* < 0.05 control vs. treatment groups, **p < 0.01 and ****p < 0.0001 vs. erastin only. One-way ANOVA, Tukey post-hoc test.

As iron accumulation is a known cause of ferroptotic cell death [14], we next investigated whether curcumin and EGCG would suppress iron accumulation resulting from erastin treatment in MIN6 cells. Intracellular iron level in MIN6 cells following exposure to erastin was 225% greater than in untreated control cells (Figure 3A). Treatment with curcumin or EGCG resulted in a decrease in erastin-induced iron accumulation in MIN6 cells. In parallel, the effect of erastin on the lipid peroxidation marker malondialdehyde (MDA) was measured. MDA levels were increased significantly following treatment with erastin, but were suppressed by co-exposure to curcumin and EGCG (Figure 3B).

### 2.6. Curcumin and EGCG Decrease Glutathione (GSH) Depletion and Glutathione Peroxidase 4 (GPX4) Degradation

**Figure 4:**
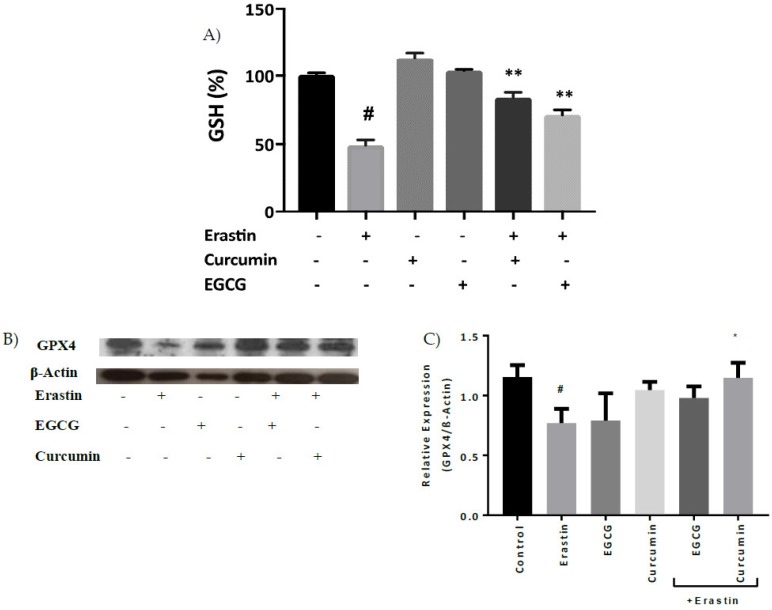
Curcumin and EGCG inhibit GSH depletion in pancreatic cells. Cells were treated overnight with 20 μM erastin in the absence or presence of curcumin or EGCG. Percentage GSH level is relative to control cell samples. (**A**) Curcumin and EGCG decreased erastin-induced GSH level. (**B**) Western blot analysis showed that curcumin alone significantly suppressed erastin-induced GPX4 level in MIN6 for 24 h. (**C**) Densitometry of Western blots GPX4 protein bands of GPX4. All the values are expressed with the mean ± SEM, *n =* 3, *^#^ p* < 0.05 control vs. treatment groups, *p < 0.05 and **p < 0.01 vs. erastin only. One-way ANOVA, Tukey post-hoc test.

GSH level was reduced in MIN6 cells exposed to erastin and co-treatment of cells with curcumin or EGCG significantly abrogated GSH decline (Figure 4A). Moreover, treatment of MIN6 cells with erastin also resulted in decreased GPX4 protein levels (Figure 4B,C), and curcumin significantly inhibited this response in the cells.

## 3. Discussion

Polyphenols offer beneficial pharmaceutical and medicinal properties [15,16] in addressing a range of ailments and disorders [16]. However, much remains unknown regarding the mechanisms of action of most polyphenolic compounds. In addition, the specific effects of therapeutic flavonoids need to be investigated [16]. Pancreatic cells are extremely susceptible to oxidative stress due to the production of high levels of endogenous ROS and a low expression of anti-oxidative enzymes [17].

The current study investigated the protective effects of different polyphenols on iron overload in a pancreatic β-cell model. The effects of iron stressors (Fenton Substrate and 8HQ+FAC, iron loading) on MIN6 cell viability were tested in vitro. FS and 8HQ+FAC exerted pronounced toxicity in the cultured cells. However, both curcumin and EGCG showed protective effects against iron-induced toxicity (Figure 1A, B). This is in agreement with the protection conferred by curcumin in brain cells cultured from Alzheimer’s patients, where curcumin inhibited cell death and decreased ROS production [18]. Moreover, Mandel et al. [19] showed a notable decrease in lipid peroxidation and oxidative modifications of membranes in neural cortex and hippocampal cells after exposure to epicatechin. The mechanisms underlying the prevention of damage by ROS and iron with curcumin and EGCG are unclear, but could involve binding of iron, prevention of redox cycling by iron, and quenching of free radicals formed by iron [20,21]. These properties could contribute to effective neutralisation of iron toxicity. 

The mechanisms of action of EGCG and curcumin are complex and likely to be multifaceted. Curcumin and EGCG are likely to modulate expression and activity of proteins of iron metabolism, suggesting that these polyphenols have abilities of an iron chelator [21,22]. Under conditions of iron deprivation, iron regulatory proteins (IRPs) are activated to repress ferritin and stabilise transferrin receptor 1 mRNA (TfR1). Activation of IRPs, translational repression of ferritin, and induction of TfR1 serve as indicators of reduced intracellular iron and are observed in cells treated with iron chelators. Both activated IRPs and TfR1 increased in response to curcumin in mice [23].

Furthermore, curcumin or EGCG co-treatment reversed cell death triggered by 20 μM erastin that was evident at 24, 48, and 72 h (Figure 2). The polyphenols also suppressed iron accumulation that was mediated by erastin treatment in pancreatic cells (Figure 3A), corroborating previous findings which demonstrated that baicalein decreased iron levels in PANC1 and BxPc3 cells against erastin-induced ferroptosis [24]. Moreover, Dixon et al. [25] showed that DFO and ferrostatin-1, potent inhibitors of ferroptosis, prevented iron accumulation and iron dependent-ROS production in breast cancer cells. 

Lipid peroxidation is one of the critical signalling events that characterize ferroptosis [25]. There is a strong relationship between iron and lipid peroxidation, as iron is known to cause ROS generation in ferroptosis [26]. Accordingly, it has been suggested that iron chelators and polyphenols might play a protective role against lipid peroxidation [24,25]. The study also demonstrated that both curcumin and EGCG exerted a decrease in MDA accumulation in pancreatic cells treated with erastin (Figure 3B). Xie et al. [24] showed that baicalein inhibited lipid peroxidation in pancreatic cancer cells treated with erastin. Furthermore, L6 skeletal muscle cells treated with EGCG and docosahexaenoic acid (DHA) exhibited lower ROS production, and lower MDA levels, than cells treated with DHA alone [27]. Together, these studies suggest that polyphenolic compounds by acting as ROS scavengers can limit lipid peroxidation of cellular membranes. Compelling evidence shows that when repair mechanisms such as antioxidant defence enzymes are overwhelmed, cell death in response to iron accumulation and lipid peroxidation becomes unavoidable. 

Reduced GSH is an essential intracellular antioxidant with a vital role in the protection of lipids and DNA from ROS [25]. In ferroptosis, erastin treatment leads to inhibition of system Xc−, which is the oxidative stress-inducible cysteine-glutamate exchange system, thus causing the inhibition of GSH biosynthesis [28,29]. Curcumin is known to induce phase II antioxidants enzymes such as glutathione reductase, glutathione-*S*-transferase, glutathione peroxidase, NAD(P)H: quinone oxidoreductase-1, and thioredoxin reductase [22]. It is shown in this study that erastin-induced GSH depletion was ameliorated by curcumin and EGCG in MIN6 cells (Figure 4A). This in turn could potentially influence the antioxidant activity of glutathione peroxidase 4 (GPX4), apart from the direct suppression of its protein level by erastin (Figure 4C) [29]. 

In agreement with the current study, breast cancer cells treated with erastin caused GSH depletion, which inactivated GPX enzymes and induced ferroptosis [30]. Interestingly, α-tocopherol complemented GPX4 deficiency and prevented cell death in GPX4 knockout mice [31]. In essence, GPX4 is one of the most important membrane protective enzymes [31]. Erastin-induced GPX4 degradation in neurons and T cells accelerated lipid peroxidation and ferroptotic cell death [32]. GSH levels decreased in the same study, but not to the level of statistical significance [32]. Consequently, erastin could bind directly to GPX4 to reduce its enzymatic activity, or indirectly reduce the level of glutathione in the cells [25,26].

The results of this study revealed that (i) curcumin or EGCG treatment reversed the cell death triggered by erastin, (ii) curcumin and EGCG ameliorated erastin-induced iron accumulation, (iii) the levels of lipid peroxidation in MIN6 cells were decreased by treatment with curcumin or EGCG, and (iv) curcumin and EGCG attenuated erastin-induced decrease in GSH and GPX4 protein levels in the cells. The study shows that curcumin and EGCG are inhibitors of ferroptosis in cultured MIN6 pancreatic β-cells and may protect against iron-induced oxidative damage in iron-overload disorders. 

## 4. Materials and Methods

### 4.1. Chemicals and Reagents

The antibodies to GPX4 and β-actin were obtained from R&D systems (Abingdon, UK) and Thermo Scientific (Dartford, UK) respectively. Curcumin, quercetin, rutin, EGCG, tannic acid and phytic acid were obtained from Sigma-Aldrich Company Ltd. (Dorset, UK) (curcumin cat. no. C7727; EGCG cat. no. E3768). Erastin was purchased from Bertin Bioreagent (Montigny-le-Bretonneux, France).

### 4.2. Cell Culture

The mouse MIN6 pancreatic β-cell line was used in this study [33]. The cells (< 30 passages) were routinely cultured in T25-cm^2^ plastic flasks. MIN6 cells were maintained in Dulbecco’s Modified Eagle’s Medium (DMEM) containing 15% heat-inactivated fetal bovine serum (FBS), 100 U/mL penicillin, and 0.1 mg/mL streptomycin. Cells were kept at 37 °C under a humidified atmosphere containing 5% CO_2_. The medium was changed twice a week. Cells were used for experimentation or split when 80–90% confluent.

### 4.3. Cell Viability Assay

Protective effects of different polyphenols against iron-induced cell death were investigated in MIN6 cells. Cellular metabolic activity was measured using the 3-(4,5-dimethyl-2-thiazolyl)-2,5-diphenyltetrazolium bromide (MTT) assay in a 96-well plate. MIN6 cells were seeded at a density of 5 × 10^4^ cells per well and pre-treated with 20 μM curcumin, quercetin, rutin, EGCG, tannic acid or phytic acid overnight, before exposure to either FS or 8HQ+FAC for 2 h. Moreover, the protective function of the polyphenols against erastin-induced ferroptosis was studied. Following this, 100 µL of fresh DMEM along with 10 μL of MTT solution (5 mg/mL in sterile phosphate buffer saline) was added to each well. After incubating for 3 h at 37 °C, 100 μL of a solubilisation buffer, dimethyl sulfoxide (DMSO) was added and incubated for 15 minutes at room temperature. To determine MTT reaction in the cells, optical density was read in a microplate reader (Bio-Tek ELx800) at 490 nm. Cell viability was expressed as a percentage of the controls [34].

### 4.4. Fenton Reaction Oxidative Stress

Optimal Fenton reaction substrate (FS) was developed and validated as 30 μmol/L FeSO_4_ and 0.5 mmol/L H_2_O_2_ [35]. Cells were pre-incubated with 20 μM curcumin, quercetin, rutin, EGCG, tannic acid or phytic acid for 24 h before exposure to FS for 2 h, to investigate the protective effect of the polyphenols against oxidative stress. Afterwards, cell viability was quantified using the MTT assay.

### 4.5. Iron-Induced Stress on Pancreatic Cells

Cells were pre-treated with various compounds before exposure to rapid iron overload damage [36] with 50 μmol/L FAC and 20 μmol/L 8HQ (8HQ+FAC). Cells were incubated for a further 2 h at 37 °C to allow rapid iron uptake. Afterwards, cell viability was determined with the MTT assay.

### 4.6. Cellular Iron Levels

Inductively-coupled plasma mass spectrometry (ICP-MS) analysis of total iron level was performed. Cell pellets collected for metal analysis by ICP-MS were re-suspended in 200 μL of 50 mM NaOH. Concentrated 68% HNO_3_ (nitric acid) was added to the samples and they were then heated for 3 h at 80 °C to complete the digestion. Measurements were made using an Agilent ICP-MS 7700 x series ICP-MS instrument under operating conditions suitable for routine multi-element analysis.

### 4.7. Lipid Peroxidation Assay

The concentration of malondialdehyde (MDA)—one of end products of lipid peroxidation—was assessed using the lipid peroxidation colorimetric assay kit purchased from Cohesion Biosciences (Slough, UK) according to the manufacturer’s instructions. 

### 4.8. Glutathione Assay 

The GSH concentration in cell lysates was assessed by using a glutathione assay kit purchased from Sigma Aldrich (Dorset, UK) according to the manufacturer’s instructions. 

### 4.9. Western Blot

MIN6 cells were lysed with RIPA Buffer (Tris/Cl (pH 7.6); 100 mmol/L, EDTA; 5 mmol/L, NaCl; 50 mmol/L, β-glycerophosphate; 50 mmol/L, NaF; 50 mmol/L, Na3VO4; 0.1 mmol/L, NP-40; Sodium deoxycholate; 0.5%), supplemented with Protease Inhibitor Cocktails (Thermo Scientific, Dartford, UK). Protein concentration was determined using Bio-Rad reagents (Bio-Rad Laboratories, Hercules, CA, USA). Twenty micrograms (20 μg) of protein extracts were loaded onto a 12% gel in SDS polyacrylamide gels (SDS-PAGE). The resolved proteins were transferred onto PVDF membranes and blocked with 5% non-fat dry milk in TBST buffer (20 mM Tris–Base, pH 7.4, 500 mM NaCl, 0.1% Tween20). The membranes were probed with primary GPX4 antibody (R&D systems, Abingdon, UK) and a β-actin secondary antibody (Thermo Scientific, Dartford, UK) diluted in TBST and incubated overnight at 4 °C. Afterwards, the membranes were probed with HRP-conjugated secondary antibody (diluted 1:5000, R&D Systems, Abingdon, UK) for 1 h at room temperature. Cross-reactivity was observed with peroxidase-linked anti-IgG by using Clarity Western ECL Substrate (Watford, UK). Densitometry of the band intensities were determined using Image J software (National Institute of Health, USA).

### 4.10. Statistical Analysis

All statistical analyses were performed using GraphPad Prism 7 (USA). Data are presented as mean ± SEM. A one-way ANOVA was used to test differences between the treatments. Comparisons between the experimental groups were conducted with Tukey’s post hoc analysis, as appropriate. Differences were considered significant at *p* < 0·05.
